# Analytical strategy for the detection of ecdysterone and its metabolites in vivo in uPA(+/+)‐SCID mice with humanized liver, human urine samples, and estimation of prevalence of its use in anti‐doping samples

**DOI:** 10.1002/dta.3032

**Published:** 2021-05-04

**Authors:** Souhail Kraiem, Maneera Y. Al‐Jaber, Hana Al‐Mohammed, Afnan S. Al‐Menhali, Noora Al‐Thani, Murad Helaleh, Waseem Samsam, Soufiane Touil, Alka Beotra, Costas Georgakopoulas, Sondes Bouabdallah, Vidya Mohamed‐Ali, Mohammed Al Maadheed

**Affiliations:** ^1^ Anti‐Doping Laboratory Qatar Doha Qatar; ^2^ Laboratoire des composés hétéro‐organiques et des matériaux nanostructurés (LR18ES11), Département de chimie, Faculté des sciences de Bizerte Université de Carthage Zarzouna Tunisie; ^3^ Centre for Metabolism and Inflammation, Division of Medicine University College London London UK

**Keywords:** 20‐hydroxyecdysone, antidoping, prevalence, supplement, uPA(+/+)‐SCID mice

## Abstract

Ecdysteroids are of interest as potential sport performance enhancers, due to their anabolic effects. The current study aimed to analyze levels of the most abundant ecdysteroid, ecdysterone (20‐hydroxyecdysone, 20‐OHE) in easily available dietary supplements, and, outline an analytical strategy for its detection, and that, of its metabolites, (1) following administration of pure 20‐OHE to uPA(+/+)‐SCID mice with humanized liver, (2) in a human volunteer after ingestion of two supplements, one with a relatively low, and the other a high, concentration of 20‐OHE, and, (3) to estimate the prevalence of use of 20‐OHE in elite athletes (*n* = 1000). Of the 16 supplements tested, only five showed detectable levels of 20‐OHE, with concentrations ranging from undetectable up to 2.3 mg per capsule. Urine of uPA(+/+)‐SCID urine showed the presence of 20‐OHE and its metabolite, 14 deoxy ecdysterone, within 24 hours (hr) of ingestion. In humans, both the parent and the metabolite were detectable within 2 to 5 hr of ingestion, with the metabolite being detectable for longer than the parent. After ingestion of a low dose supplement, the parent and metabolite were detectable for 70 and 48 hr, while following the higher dose it was 96 and 48 hr, respectively. Analysis of urines from athletes (*n* = 1000) confirmed four positives for 20‐OHE, suggesting a prevalence of use of 0.4%. Prevalence of its use by elite athletes was relatively low, however, this needs to be confirmed in other populations, and with other related ecdysteroids.

## Graphical abstract

Ecdysteroids have recently been included in the WADA Monitoring program due to their anabolic effects. Concentrations of 20 OHE in dietary supplements are variable. Urinary 20‐OHE and 14‐deoxy‐20‐OHE were detected in uPA(+/+)−SCID mice within 24 hours (hr) of ingestion. In humans, 20‐OHE and its metabolite were detectable for 70 and 48 hr (2 caps of 0.0088 mg) and for 96 and 48 hr (two capsules of 2.3 mg). In elite athletes there was a prevalence of use of 0.4%.

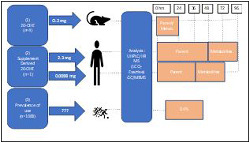

## 1 INTRODUCTION

Ecdysteroids are commonly known as arthropods steroid hormone for molting, metamorphosis, embryogenesis and reproduction.^1^ They are also identified in some plants species, which can facilitate its extraction and consumption.^2^, ^3^, ^4^ Various pharmacological properties of ecdysteroids are recognized, specifically their ability to enhance physical performance through beneficial changes to body composition and anabolic effects. The anabolic effects of ecdysteroids were reported to be mediated by activation of beta estrogen receptor.^4^ Thus, it can be considered as a perfect candidate for the use by athletes.^5^, ^6^, ^7^, ^8^ As a result, it was recently included in the WADA monitoring program of 2020.^9^

**FIGURE 1:**
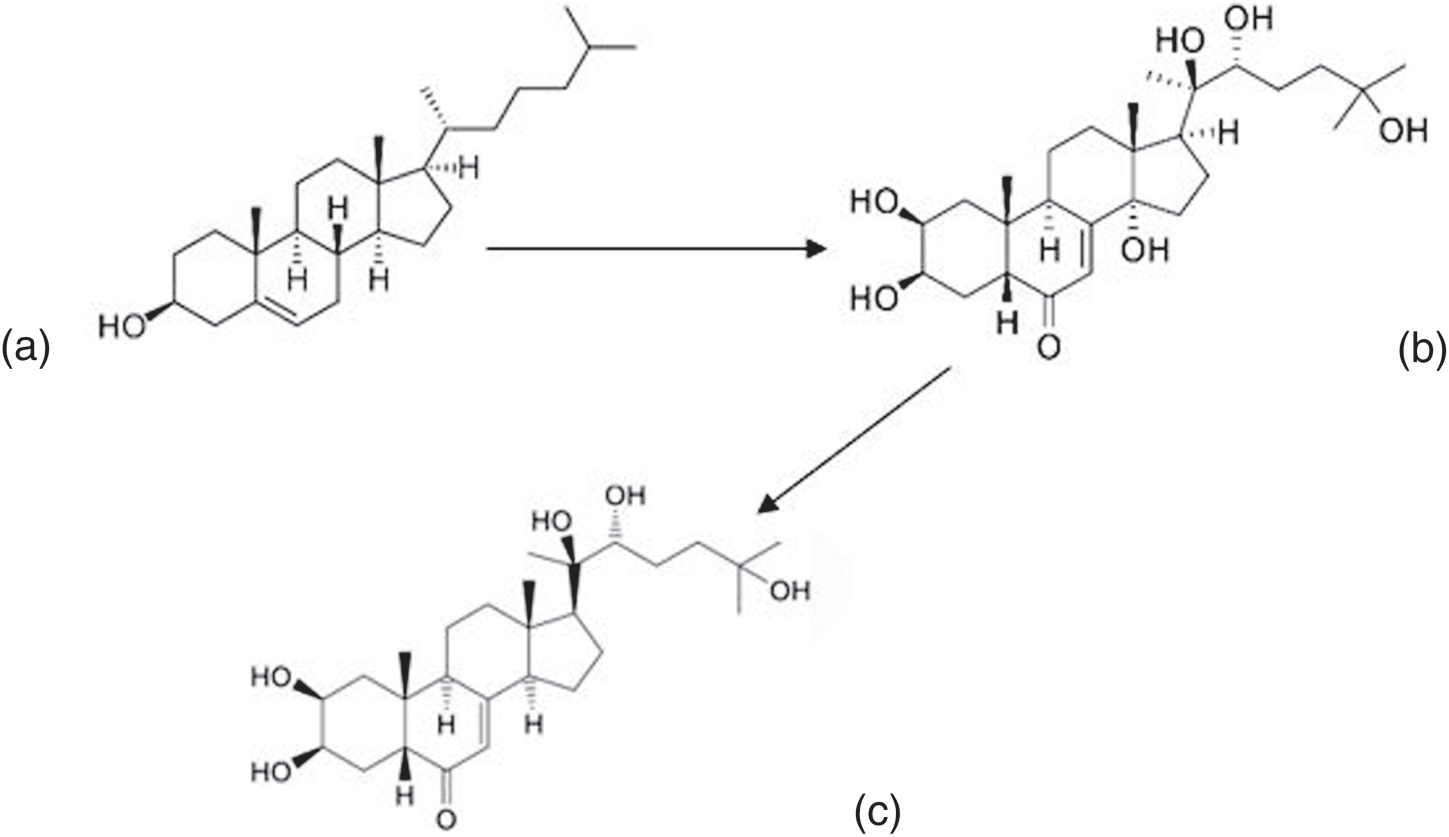
Chemical structure of cholesterol (a), 20‐OHE (b) and 14‐deoxy‐20OHE (c)

There are different classes of ecdysteroids including, ecdysterone, ecdysone and turkesterone2‐3, among which 20‐hydroxyecdysone (ecdysterone, 20‐OHE) is the most abundant and the most studied. The chemical structure of ecdysterone is a 27‐carbon (C27) molecules derived from cholesterol(C_27_H_46_O) (Figure 1).

Ecdysterone(C_27_H_44_O_7_) (2β,3β,14α,20β,22R,25‐hexahydroxy‐5β‐cholest‐7‐en‐6‐one) is an ingredient that is added in some commercial products, such as dietary supplements with claims of abilities to enhance physical performance. Few studies are available in the recent literature on the metabolism of ecdysterone. The breakdown of ecdysterone differs considerably between species, generating different metabolites. For example, in human urine excretion of deoxy‐ecdysterone metabolites were reported as 2‐deoxyecdysterone, deoxyecdysone and 14‐deoxy‐ecdysterone;^10^, ^11^ in calf urine, 14‐deoxy‐20‐hydroxyecdysone, 20,26‐dihydroxyecdysone and 14‐deoxy‐20,26‐dihydroxyecdysone;^12^ and in mice/rat excretion: 14‐deoxy‐20‐hydroxyecdysone, poststerone and 14‐deoxypoststerone.^7^, ^13^ The inclusion of ecdysteroids on WADA monitoring list has regenerated interest in investigating its metabolism and prevalence of use in humans. The detection of 20‐OHE and its metabolites after administration of high doses in human volunteers (20 and 50 mg) has been reported by Tsitsimpikou et al.^10^ and Parr et al,^11^ respectively. However, the doses present in the supplements vary considerably and often do not accurately reflect those on the label.^14^ Therefore, there is a need to quantitate the 20‐OHE levels in the supplements themselves, as well as, detect the fate, both parent and metabolite, after in vivo ingestion.

Thus, aims of the present study were to investigate the analytical strategy for the detection of 20‐OHE, and its metabolites: (1) following administration of pure ecdysterone to uPA(+/+)‐SCID mice with humanized liver; (2) in a human volunteer after ingestion of two supplements with low and high concentrations of 20‐OHE; and (3) to investigate the presence of 20‐OHE in anti‐doping samples to estimate prevalence of use by elite athletes.

## 2 EXPERIMENTAL

### 2.1 Supplements, reagent and chemicals

All reagents and solvents used for the present work were of analytical and HPLC grade and purchased from several suppliers. Reference standard of 20‐OHE was from Sigma‐Aldrich, Chemie (Steinheim, Germany). The standard solution of 20‐OHE (stock and working solutions) was prepared in methanol. The stock solution (1 mg/ml), was diluted to working solution of 100 and 1 μg/ml and stored at −20°C. The internal standard used for the LC MS analysis was 17a‐Methyltestosterone (50 μg/ml), whereas for the GCMS Analysis was a mixture of d3‐Testosterone (4 μg/ml), d3‐epitestosterone (4 μg/ml), d4‐androsterone glucuronide (43.7 μg/ml), d5‐ethiocholanolone (50 μg/ml), d5‐5B‐Androstane‐3a,17B‐diol (5 μg/ml) and 17a methyltestosterone (50 μg/ml).

Sixteen dietary supplements were openly sourced through the internet (amazon.com, USA). Table 1 shows names of the supplements and declaration on the label about the presence of ecdysterone. Methanol, sodium hydrogen carbonate, potassium carbonate, dithioerytritol (DTE) were obtained from Sigma Aldrich (Darmstadt, Germany). β‐glucuronidase from *E. coli* was from Roche Diagnostics GmbH (Mannheim, Germany), Diethyl ether from Merck (Darmstadt, Germany) while MSTFA was from Roche (Mennheim, Germany).

**TABLE 1:** Concentration of 20‐hydroxyecdysone in various supplements

S.No	Name	Label claim	Concentration	
			mg/capsule	mg/g^a^
1	ZMA PM	Zinc, magnesium & Vit B6	not Detected	not Detected
2	Beta‐X	Carnosyn Beta‐Alanine	not Detected	not Detected
3	Ecdybolin	Ecdysterone (20‐hydroxyecdysone)	1.008	1.5
4	Suma root	400 mg(pfaffia paniculata) also contains ecdysterone	0.176	0.13
5	Norateen II	Beta Ecdysterone	Not Detected	Not Detected
6	Desire X	Hydroxyecdysone(20‐hydroxyecdysone)	0.0088	0.014
7	Z‐Force	Zinc, magnesium & Vit B6	Not Detected	Not Detected
8	Bio pro plus	bio identical thymic proteins & zinc	Not detected	Not detected
9	MSB methyl plus	Pyridoxal‐5‐Phosphate (P5P) (active form of B6) on testosterone.	Not detected	Not detected
10	Power meal	indirect effect for the Alpha‐lipoic acid on testosterone	Not detected	Not detected
11	Immunectar	source of Beta‐ecdysteron	0.0004	0.0005
12	Natural Sterol extreme	Beta Ecdysterone	Not detected	Not detected
13	Animal Stack (USA)	Zinc, magnesium & Vit B6	Not detected	Not detected
14	Promax Extreme	protein and glutamine has direct effect on testosterone	Not detected	Not detected
15	Medicinal cyathula root	no clear information	Not detected	Not detected
16	Turkesterone	Turkesterone	2.3	1.95

^a^ Five capsules pooled together then two aliquots of 0.5 mg were taken for analysis and injected in triplicates, the presented concentration represents the average of six determinations.

### 2.2 Supplement extraction

The contents of five capsules were mixed and two aliquotes of 0.5 gm each were taken for the extraction. Each aliquote was dissolved in 5 mL of carbonate buffer (pH 9‐10) by shaking for 30 min. Thereafter, 5 ml of diethyl ether was added and shaking continued for another 30 min. The mixture was then centrifuged at 2602*g* for 10 min to separate the organic layer, which was evaporated under nitrogen flow. The pellet was reconstituted in 200 μL of mobile phase (80/20 (solvent A/solvent B), the mobile phase consisted of water (solvent A) and Acetonitrile/Water(90:10) (Solvent B), both containing 5 mM ammonium formate and 0.02% formic acid). Each reconstituted sample was injected in triplicate and analyzed for 20‐OHE.

### 2.3 Sample preparation

#### 2.3.1 SCID mice urine samples

uPA(+/+)‐SCID mice (male), transplanted with primary human hepatocytes (chimeric mice) at KMT Hepatech lnc. (Edmonton, Canada) were used for experiments. All in vivo experiments with these animals were carried out at KMT Hepatech lnc, as per approval of the National Laboratory Ethics Committee (ECD06/09). The non‐chimeric mice, which were not transplanted with human hepatocytes, served as the controls. Prior to administration of drug, human albumin concentration in the mouse plasma was determined to confirm successful transplantation. Chimeric mice with elevated human albumin were used in the study. The chimeric mice (*n* = 8) were treated with daily doses of 20‐OHE (0.2 mg, *n* = 4) or vehicle (5% ethanol/PBS, *n* = 4). Similarly, non‐chimeric mice (*n* = 2) were also treated with 20‐OHE (0,2 mg, *n* = 1) or vehicle (5% ethanol/PBS, *n* = 1). 20‐OHE was administered by oral gavage on days 0, 1 and 2 of the experiment. Urine samples were collected noninvasively over a 24‐hr period, on days 0 (prior to), and on days 1, 2, 3 and 4 after 20‐OHE administration, and stored at −80°C until shipped to the Anti‐Doping Lab Qatar (ADLQ) on dry ice for analysis.

#### 2.3.2 Human urine samples

After identifying the presence of 20‐OHE in five out of 16 supplements, (Table 1), two supplements, one with low (Desire X 0.0088 mg/capsule) and another with high levels (Turkesterone 2.3 mg/capsule) were selected for the excretion studies.

Two capsules (recommended dose) of Desire X were administered to one healthy male volunteer (43 years, 90 kg) who declared not to have used any nutritional supplement or medication during the preceding 3 months. A blank urine sample was collected prior to administration.

After 45 days, the same volunteer was administered two capsules (recommended dose) of Turkesterone. A blank urine was collected preadministration. Following administration all the urines were collected for the first 3 days, with only the morning urine being collected for further 3 days, as 20‐OHE has previously been shown to be rapidly eliminated within the first 24 hr. All the urine samples were stored frozen at −20°C until analysis.

#### 2.3.3 Urine sample preparation

##### SCID mice urine

0.4 mL of urine was spiked with 50 μl of internal standard as detailed in 2.1 above and 0.25 ml of phosphate buffer (pH 7) was added. Enzymatic hydrolysis was performed by adding 25 μl of β‐glucuronidase from *E. coli*. The mixture was incubated for 60 min at 55°C. After hydrolysis, the pH was adjusted between 9 and 10 with liquid carbonate buffer. A liquid–liquid extraction was performed by adding 5 ml of diethyl ether. After centrifugation at 1328*g* for 5 min, the organic layer was separated from the aqueous phase by freezing and evaporation under nitrogen at 50°C. The remaining residue was reconstituted in 100 μL of mobile phase as detailed in 2.2 and 5 μl were injected to the UHPLC/HRMS system. Additionally, for GC/MSMS analysis, a derivatization step was performed to the dry residue by adding 50 μL of MSTFA/NH4I/DTE (1000/1/2: v/w/w) and incubating for 1 hr at 65°C followed by irradiation in the microwave (700 w 90 s).

##### Human urine

2.5 ml of urine was spiked with 25 μl of internal standard as detailed in 2.1 above and 0.25 ml of phosphate buffer (pH 7.0) was added. The enzymatic hydrolysis was performed by adding 50 μl of β‐glucuronidase from *E. coli* and incubated at 55°C for 1 hr. After hydrolysis, 0.30 ml of carbonate buffer was added and extraction was performed with 5 ml of diethyl‐ether by shaking for 20 min. After centrifugation, the organic layer was separated by freezing the aqueous phase and evaporated to dryness under nitrogen flow. The dry extract was derivatized by adding 50 μl of a mixture of MSTFA/NH4I/DTE (1000/1/2: v/w/w) and incubating at 65°C for one h followed by microwave irradiation (700 w, 90 s).

### 2.4 Prevalence study

Prevalence of 20‐OHE use was determined in anti‐doping urine samples destined for discard, as per WADA ISL clause 5.3.3.1.^15^

### 2.5 Instrumentation

#### 2.5.1 LC‐QQQ equipment

Separation of ecdysteroid was performed on Agilent 1200 HPLC chromatographic system with a mobile phase solvent A (100% water containing 5‐mM ammonium formate in 0.02% formic acid)/B (Acetonitrile/water [90:10 v/v] containing 5mM ammonium formate and 0.02% formic acid) (v/v). A gradient elution program (A) and (B), was employed at a constant flow rate of 0.2 ml/min. The analysis run time is 10.5 and 4 min for the equilibrium time (post Run), the injection volume was 5 μl. A triple quadrupole mass spectrometer (Agilent 6,490, Palo Alto, CA, USA) with an ESI source in the positive ion mode was used with a drying gas flow of 15 L/min at 250°C, sheat gas flow of 11 L/min at 250°C the desolvatation temperature was set to 250°C and the nebulizer gas to 45 psi. The capillary voltage was 4 kV and the fragmentor voltage was fixed at 380 V, the parent compound and its metabolite were detected using the Multiple reaction monitoring (MRM) acquisition mode.

#### 2.5.2 LC/QExactive Orbitrap

UHPLC/HRMS conditions. A Dionex UHPLC system (Thermo Scientific) was used for the chromatographic separation. The system consisted of a vacuum degasser, a high‐pressure binary pump, an autosampler with a cooled sample tray and a column oven. Chromatographic separation was performed at 40°C using a Zorbax Eclipse Plus C18 column (100 × 2.1 mm i.d., 1.8‐mm particle size; Agilent Technologies). A gradient elution program was employed at a constant flow rate of 0.2 ml/min. The analysis run time was 10.5 min and the injection volume was 3 μl.

The high‐resolution mass spectrometry analysis was performed using a QExactive Orbitrap‐based mass spectrometer (Thermo Scientific) operated in the positive–negative polarity switching mode and equipped with a heated electrospray ionization (HESI) source. Source parameters were: sheath gas (nitrogen) flow rate, auxiliary gas (nitrogen) flow rate and sweep gas flow rate: 40, 10 and 1 arbitrary units respectively; capillary temperature was set at 300°C, heater temperature at 30°C and spray voltage at +4.0 and −3.8 kV, for the positive and negative ionization, respectively. The instrument operated in full scan mode from m/z 100 to 1000 at 17,500 resolving power for both polarities. The automatic gain control (AGC) was 10 E6.

#### 2.5.3 GC QTOF‐MS

Analysis was performed using an Agilent GC 7890 coupled with an Agilent 7200 QTOF MS equipped with HP‐5MS 5% phenylmethyl siloxane capillary column (30‐m length, 250 μm ID, 0.25 μm film thickness), the quadrupole device prior to TOF MS analyzer provides the capability of applying MS/MS experiments. Helium was used as carrier gas with a constant flow set at 1.1 ml/min. The injection volume was 1 μl in a split mode of (10:1). The injection port and the interface temperatures were set at 280°C. The oven temperature program consisted of the following temperatures: 160°C for 0 min, ramped at 10°C /min to 200°C, then ramped at 2°C/min to 220°C, ramped at 6°C/min to 292°C, 50°C/min up to 310°C and held for 10 min, total run time 36.36 min. Electron impact ionization (EI) at 70 eV was used for compound ionization with source temperature of 250°C. 2 GHz extended dynamic range (EDR) acquisition mode was used for TOF data acquisition. The applied MS range (50–1050 Da) allows measurement of typical large molecules analyzed by the GC/QTOF.

#### 2.5.4 GCQQQ‐MS

An Agilent (Agilent Technologies, Palo Alto, CA, USA) GC 7890 gas chromatograph coupled with an Agilent 7000B triple quadrupole mass spectrometer equipped with 5% Phenyl polysilphenylene‐siloxane capillary column (30 m length, 0.25 mm ID, 0.1 μm film thickness, SGE BP X5) was used. The oven temperature program consisted of following temperatures: 160°C for 0 min, ramped at 10°C/min to 200°C, then ramped at 2°C/min to 220°C, ramped at 6°C/min to 292°C and at 50°C to a final temperature of 310°C (held for 2 min, runtime 28.36 min). The transfer line was set at 280°C. Helium was used as a carrier gas at a flow rate of 1.0 mL/min. 2 μL was injected into GCQQQMS using a split mode at a ratio of 1:10 with injector temperature at 280°C, the source temperature was set at 230°C. In the QqQ collision cell Helium was used as a quench gas at 2.25 ml/min and N2 as a collision gas at 1.5 ml/min.

### 2.6 Statistical analysis

**FIGURE 2:**
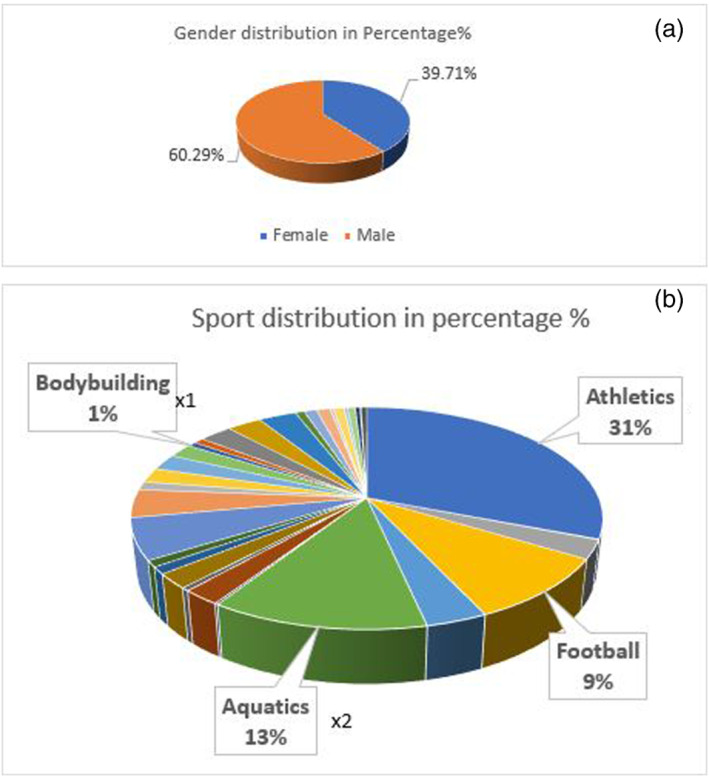
Sample distribution according to gender (a) and sports (b) [Colour figure can be viewed at wileyonlinelibrary.com]

No detailed statistical analysis could be carried as only information on sports discipline and gender were available on these athletes. Therefore, the data are shown as a Venn diagram depicting the various disciplines, and also shows the sport in which the positive results were obtained. All basic statistics were carried out with Excel (Figure 2).

## 3 RESULTS AND DISCUSSION

### 3.1 Quantitation of 20‐OHE in dietary supplements

From a total of 149 supplements cited by Lafont & Dinan,^2^ 15 dietary supplements which were openly sourced through the internet were analyzed for the presence of 20‐OHE. In addition, Turkesterone supplement was also analyzed. Seven supplements, as per label claim, were supposed to contain 20‐OHE. However, only five of these were found to have detectable levels of the ecdysteroid. Furthermore, their concentrations varied significantly (Table 1). The concentration of 20‐OHE is given in both as mg/capsule and as mg/g.

The lowest quantity (0.0005 mg/g) was found in Immunectar, while the highest quantity (1.5 mg/g) was present in the Turkesterone supplement, which despite its name, contained 20‐OHE. For further studies, Desire X (0.014 mg/g) and Turkesterone (1.5 mg/g) were chosen in order to investigate the variation in the urinary excretion after low and high doses of supplement‐derived 20‐OHE. The limit of detection (LOD)of the method was 10 ng/g.

The recently reported study of Ambrosio et al.^14^ showed variability of the 20‐OHE concentration in 12 supplements tested and also mentioned the fact that the concentration of 20‐OHE was often significantly lower than claimed on the label. The current study confirmed that finding, albeit in different supplements. However, their study did not name the exact supplements, but used study code numbers.

### 3.2 Detection and identification of 20‐OHE and its metabolite

#### 3.2.1 SCID mice

**FIGURE 3:**
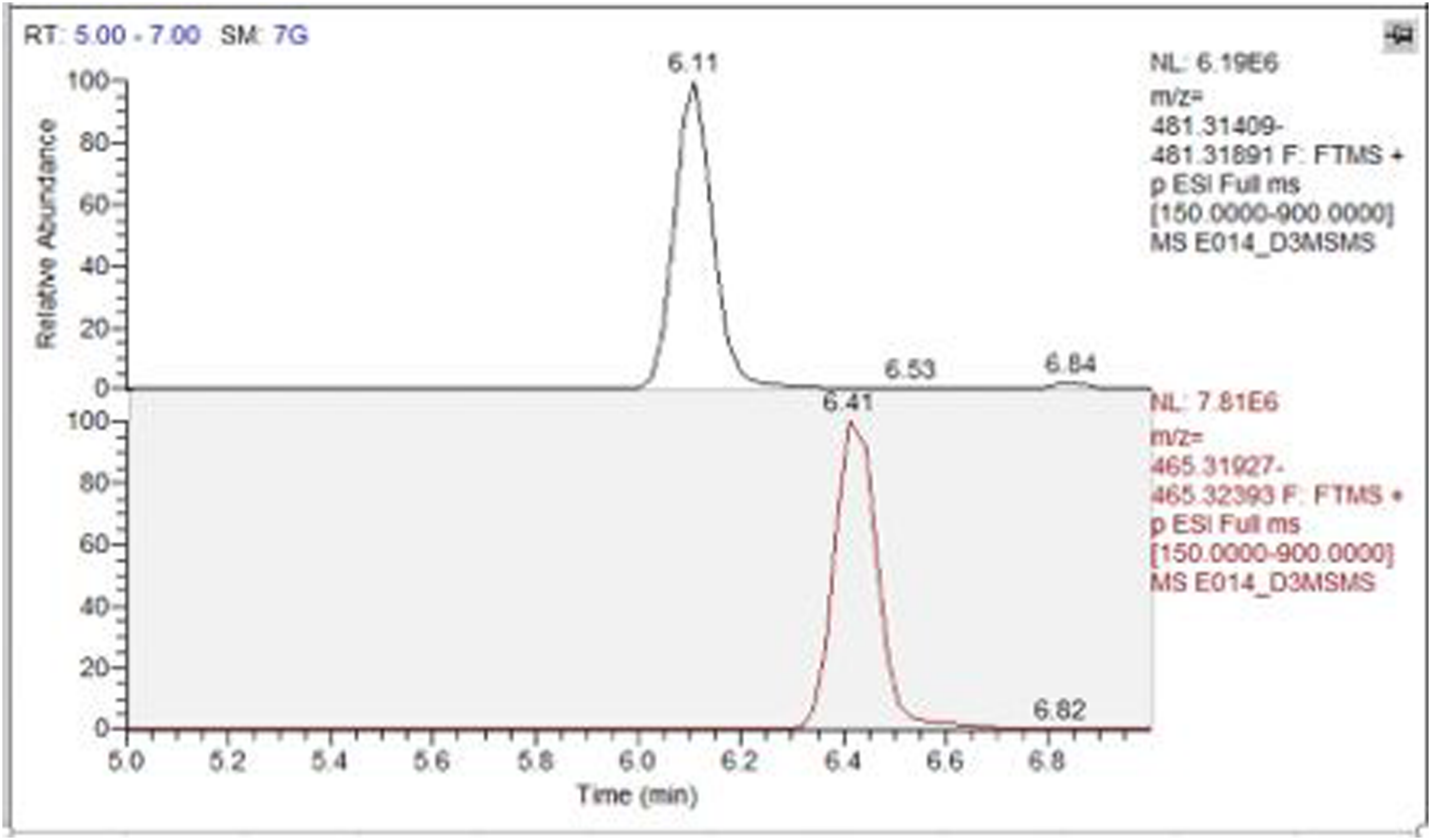
LC (ESI+) HRMS full scan chromatograms of the extracted ions for 20‐OHE (481 m/z) and 14‐deoxy‐20‐OHE (465 m/z) [Colour figure can be viewed at wileyonlinelibrary.com]

The analytical strategy adopted for the detection of 20‐OHE and its metabolite was on LCMS QQQ, LCMS/QExactive, GCMS QQQ and GCMSQTOF. 20‐OHE and its metabolite, 14‐deoxy‐20‐OHE, were detected in all urine samples collected from chimeric (*n* = 4) SCID mice treated with 0.2 mg of 20‐OHE, but not in those treated with vehicle. In LCMS/QExactive Orbitrap, the parent compound was eluted at 6.11 min whereas the metabolite 14‐deoxy‐20‐OHE was eluted at 6.41 min (Figure 3).

**FIGURE 4:**
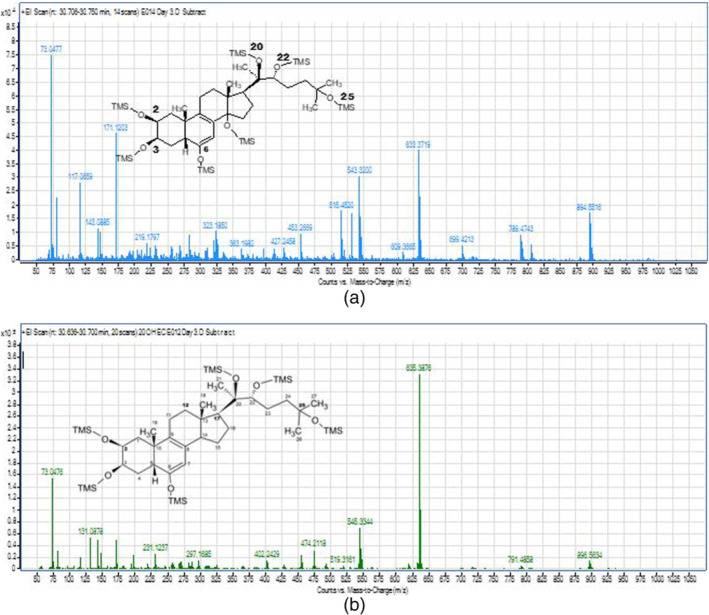
(a) GCMSQTOF full scan spectrum of the parent compound 20‐OHE showing the specific TMS fragments (894.5516 m/z [M^+^‐OTMS] and the TMS fragment 633.3719 m/z) in SCID mice urine. (b) GCMSQTOF full scan spectrum of 14‐deoxy‐20‐OHE showing the specific TMS fragments (896.5634 m/z [M^+^‐OTMS] and TMS fragment 635.3876 m/z) in SCID mice [Colour figure can be viewed at wileyonlinelibrary.com]

The GCMS QQQ data showed two abundant peaks in full scan mode, the parent compound with specific ions m/z 894 resulting from loss of OTMS from the molecular weight after TMS derivative m/z 984. The most abundant fragment for the parent compound 20‐OHE is m/z 633 and successive elimination of OTMS leading to m/z 543, 453, 363. The parent compound 20‐OHE exhibit a standard fragmentation and characterized the presence of side chain C‐20 to C‐25, such us m/z 171, 131, 261, 81. The GCMSQQQ data of 14‐deoxy‐20‐OHE showed the specific ions of m/z 896 as molecular ion with TMS derivatives, The most abundant fragment at m/z 635 and successive eliminations of OTMS leading to m/z 544, 455, 365 and the same fragments of indication of the presence of the side chain. The GCMSQTOF high resolution data showed the same fragments for both molecules (Figure 4a,b).

**FIGURE 5:**
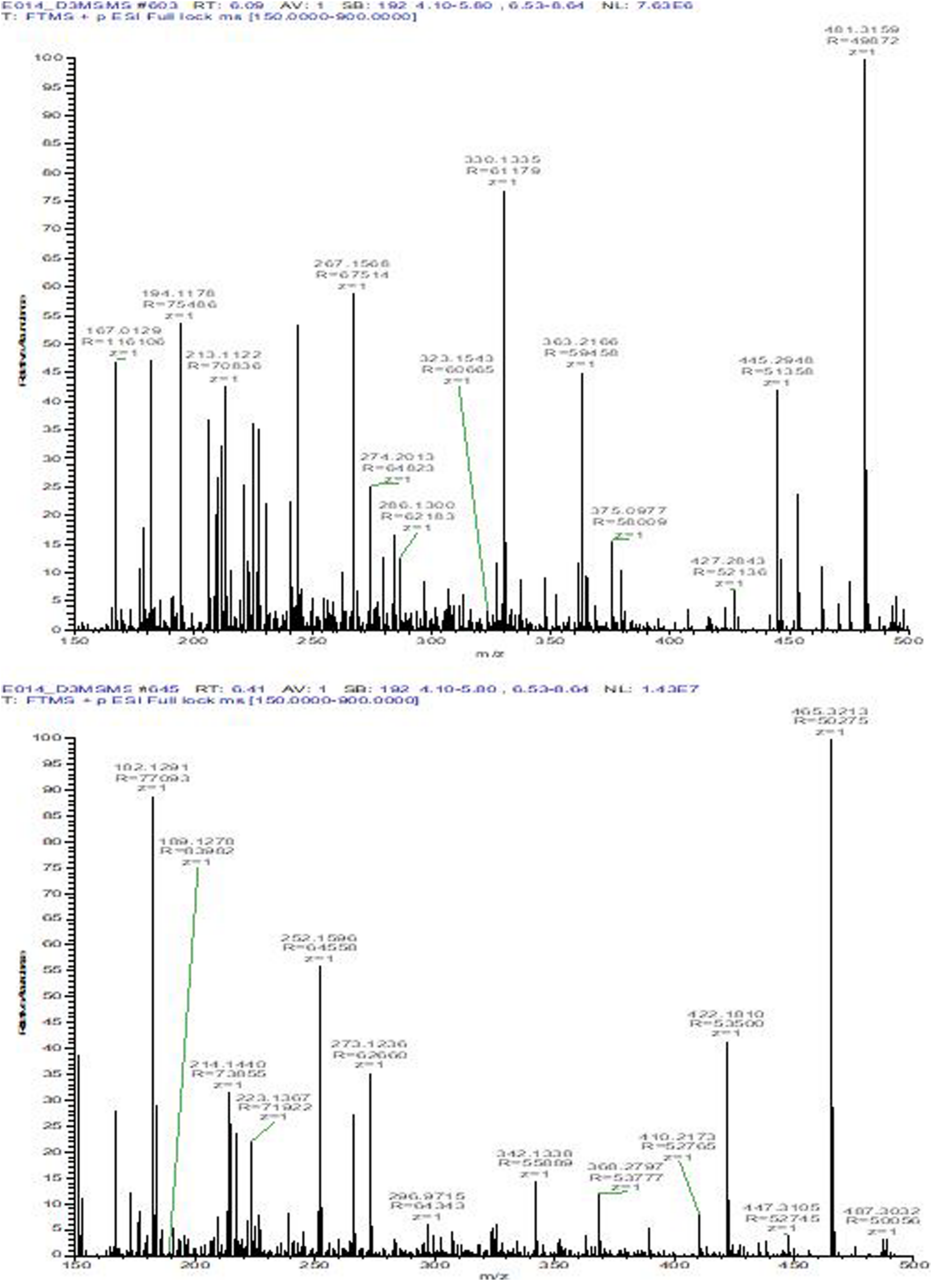
Full scan spectrum of 20‐OHE (upper) and 14‐deoxy‐20‐OHE (lower) by ESI(+)‐LC HRMS showing the molecular ion at 481.3160 (M + H)^+^ and 465.3211(M + H)^+^ respectively [Colour figure can be viewed at wileyonlinelibrary.com]

Due to the polarity difference between the parent and metabolite, the metabolite is eluted before the parent compound, because after derivatization more TMS reacting with the hydroxyl group and/or the keto group more retained and interact with the stationary phase of the column, which is also explained by Parr et al.^11^ As per the theoretical molecular weight of 20‐OHE, which is m/z 480.3087, and that of metabolite, which is m/z 464.3137, the LC‐(ESI+) HRMS full scan analysis showed (M + H)^+^ at m/z 481.3170. and (M + H)^+^ at m/z 465.3216 respectively Figure 5.

In contrast with GCMS analysis, LCMS analysis showed elution of metabolite after parent compound, since the analysis was carried out with C18 column, because the more polar compound is less retained. The elution order in both LCMSMS and GCMSMS was affected based on the polarity, due to the presence of hydroxyl groups in 20‐OHE (at positions 2, 3,14, 20, 22 and 25) and its metabolite (at positions 2, 3, 20, 22 and 25), and keto group (in position 6) for both parent and metabolite. For GCMSMS the molecules needed to be derivatized for both the hydroxyl groups and the keto group to be analyzed.^16^ In addition to conventional derivatisation with a mixture of MSTFA, catalyst and the antioxidant, a microwave assistance reaction was used to overcome incomplete derivatisation. The power and the time were optimized and it was observed that 90 s at 700 w was the optimum condition for a complete reaction for both parent drug and metabolite. The difficulty in the derivatisation was also explained by Tsitsimpikou et al.^10^ and Parr et al.^11^

**FIGURE 6:**
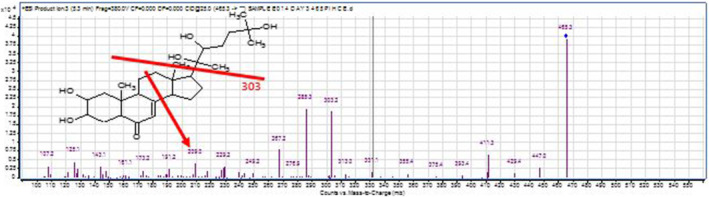
Product ion scan spectrum LCMS QQQ (precursor(M + H)^+^ = 465.3 m/z for 14‐deoxy‐20‐OHE in SCID mice urine) [Colour figure can be viewed at wileyonlinelibrary.com]

Based on mass spectrometry the presence of the deoxy ecdysterone metabolites was investigated, either 2‐deoxy or 3 deoxy and 14‐deoxy‐ecdysterone, with no indication of the presence of Ponasterone. A 14‐deoxy‐ecdysterone generated m/z 303 (C19H27O3)^+^ as a fragment, resulting from a cleavage of the side chain with three hydroxyl groups at positions 20, 22 and 25. Based on that observation a precursor ion scan was performed in order to elucidate the parent mass which leads to that fragment Figure 6.

**FIGURE 7:**
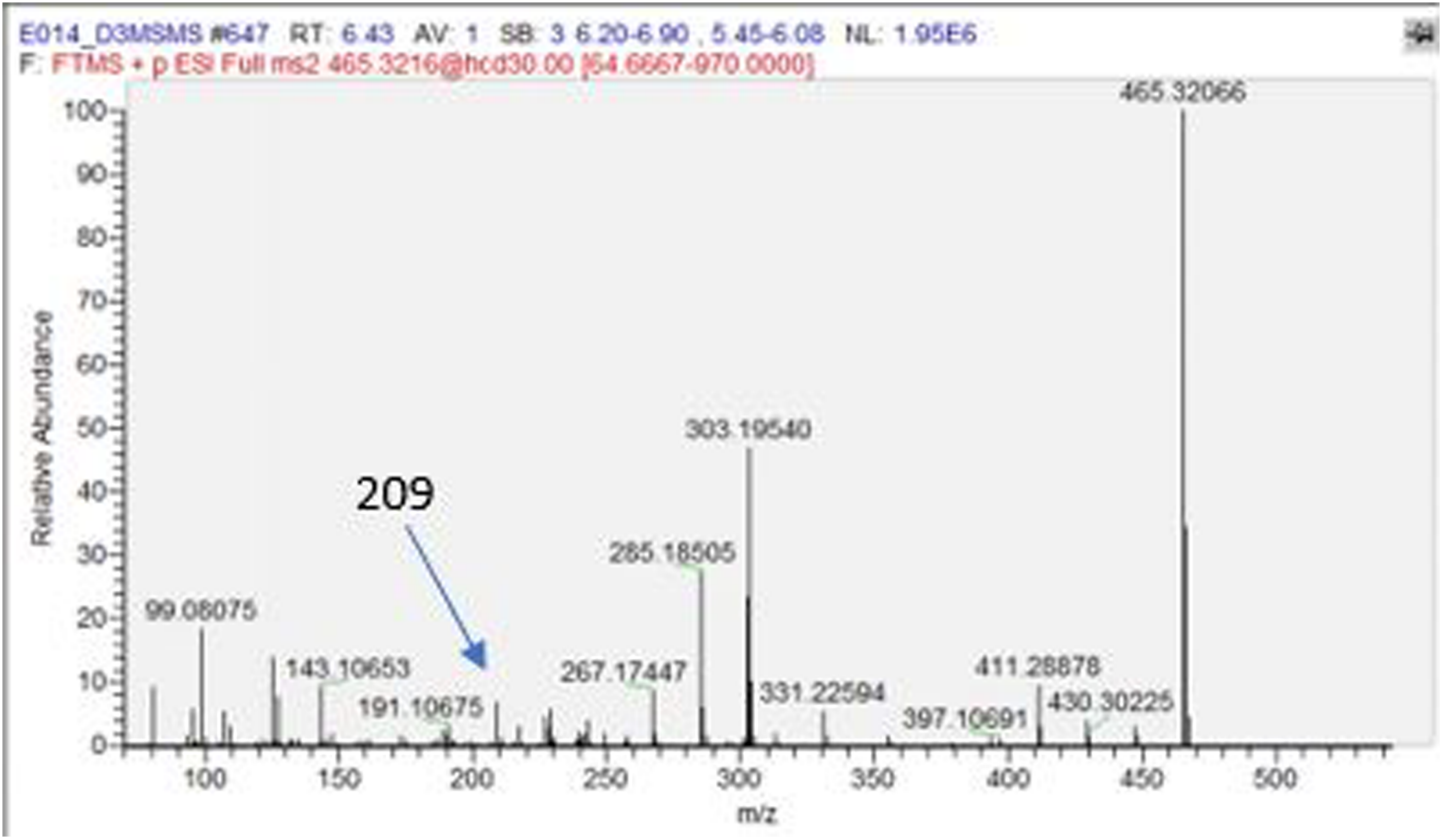
LCMSMS,(ESI+)‐HRMS spectrum for 14‐deoxy‐20‐OHE in SCID mice urine[M + H]^+^ = 465.3211 mass accuracy 2 ppm [Colour figure can be viewed at wileyonlinelibrary.com]

The same was performed for the ion m/z 285 (C_19_H_25_O_2_^+^) resulting from a loss of water molecule from m/z 303 fragment. Additionally, the LCMSMS high resolution performed on molecular mass m/z 465.3211 produced the same fragments (m/z 285.1850 and m/z 303.1954) with a mass error less than 1 ppm (Figure 7).

Furthermore, the full scan spectrum by LCMS shows an ion at m/z 209 resulting from the cleavage of C11‐C12 and C8‐C14 confirming that two hydroxyl group are in positions 2 and 3 of the molecule. On GCMSQTOF, after derivatisation 14‐deoxy‐ecdysterone is eluted just before the parent compound at 30.48 min in full scan whereas the parent compound is eluted at 30.55 min. Besides the specific fragments for ecdysteroids resulting from the side chain such as m/z 171.1200, m/z 131.0891 and m/z 896.5564 as a molecular ion two specific ions are generated m/z 231 resulting from a fragmentation of C1‐C10 and C4‐C5 whearas m/z 217 fragment resulting from a fragmentation of C1‐C10 and C3‐C4 which consolidate the hypothesis of two hydroxyl groups at positions 2 and 3. Figure 4b.

**FIGURE 8:**
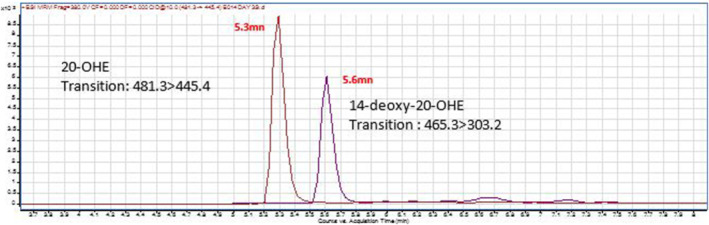
LCMS QQQ overlaid extracted MRM ion chromatogram for the selected transitions of 20‐OHE(481.3 > 445.4) and its metabolite 14‐Deoxy‐20‐OHE(465.3 > 303.2) eluted at 5.3 and 5.6 min, respectively [Colour figure can be viewed at wileyonlinelibrary.com]

Taking in to account the above constatations, an LCMS QQQ method was developed in order to detect the parent compound and its 14‐deoxy metabolite. The Figure 8 shows chromatographic separation for 20‐OHE and metabolite, 14‐deoxy‐ecdysterone eluted at 5.3 and 5.6 min respectively.

**FIGURE 9:**
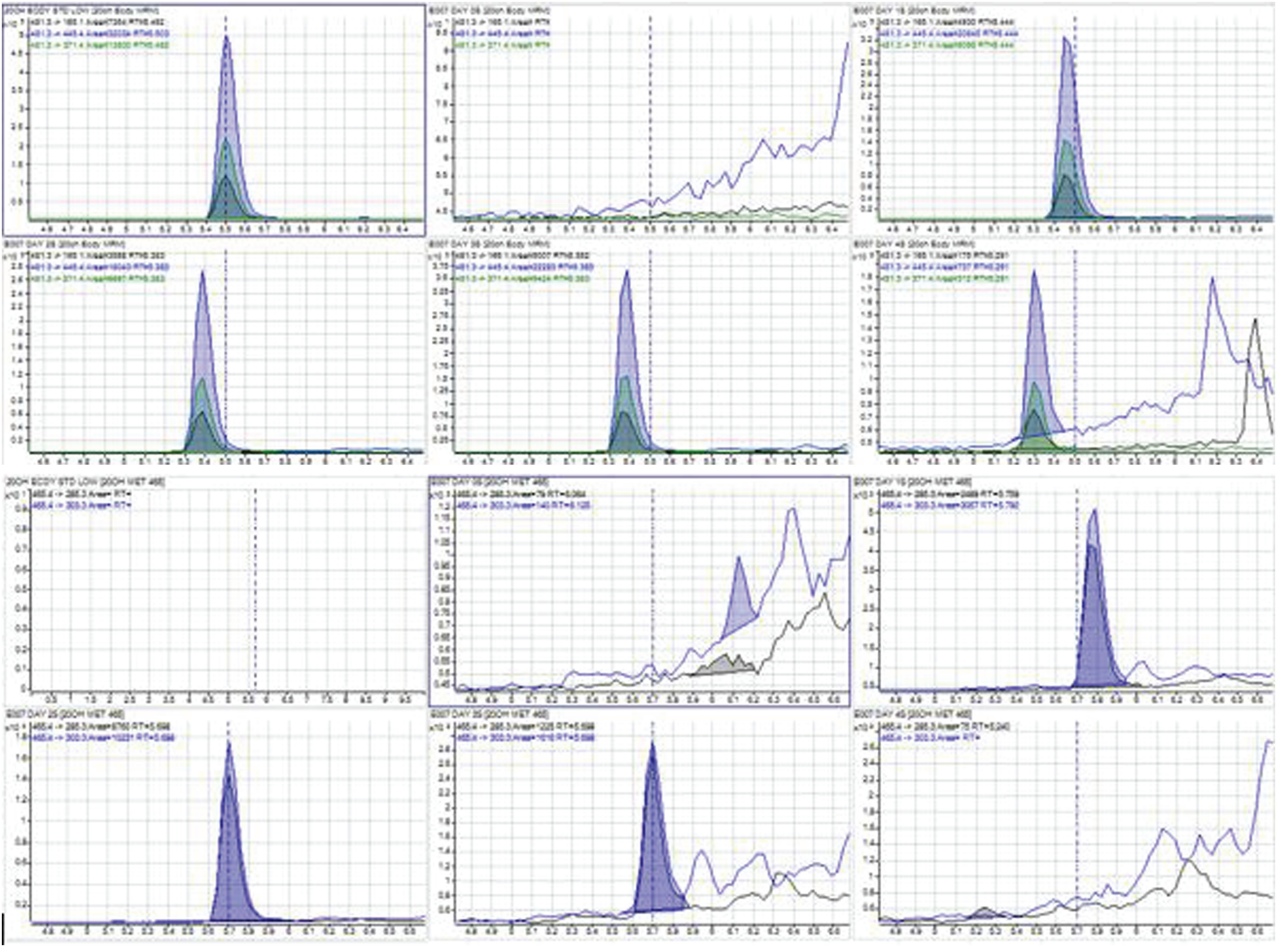
(a) LCMSMS chromatograms of 20‐OHE (day 0, 1, 2, 3 and 4) of SCID mice urine. (b) LCMSMS chromatograms of 14‐deoxy‐20‐OHE (day 0, 1, 2, 3 and 4) of SCID mice urine [Colour figure can be viewed at wileyonlinelibrary.com]

The parent compound was detectable up to 48 hr after the ingestion while the metabolite was detectable up to 24 hr (Figure 9a,b).

#### 3.2.2 Human excretion studies

Based on mass spectrometry data, both high and low resolution, the 20‐OHE metabolite 14‐deoxy‐20‐OHE was tentatively identified, as described above in SCID mice samples. A GC/MS/MS method was developed for the detection of 20‐OHE and its metabolite, 14‐deoxy‐20‐OHE in the excretion study performed with two different supplements. The monitored transitions are reported in Table 2.

**TABLE 2:** MRM acquisition parameters for identification of 20‐OHE, 14‐Deoxy‐20‐OHE, ponasterone A and internal standard (17α methyltestosterone) in screening procedure by GCMS QQQ

Compound	Precursor ion(m/z)	Product ion (m/z)	Retention time (min)	Collision energies(v)
20‐OHE	633	543, 453	26.88	15, 15
14‐Desoxy‐20 OHE	635	545, 455	26.17	20, 15
Ponasterone A	806	716, 633	25.8	10, 15
17αMethyltestosterone	446	301.2	18.2	20

**FIGURE 10:**
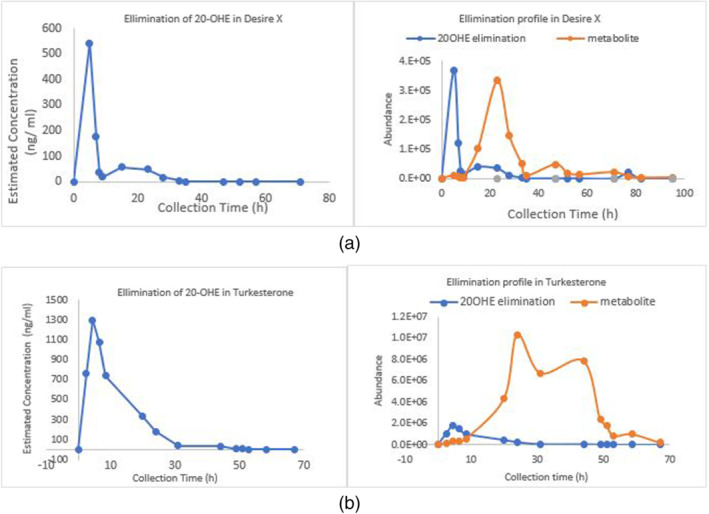
(a) Urinary excretion profile (GCMS QQQ) of 20‐OHE and its main metabolite 14‐deoxy‐20‐OHE after ingestion of two capsules of desire X supplement. (b) Urinary excretion profile (GCMS QQQ) of 20‐OHE and its metabolite 14‐deoxy‐20‐OHE after ingestion of two capsules of turkesterone supplement [Colour figure can be viewed at wileyonlinelibrary.com]

With the administration of a single oral dose of Desire X (0.0088 mg/capsule) and Turkesterone (2.3 mg/capsule), which was 2 capsules for both, the parent compound and its metabolite, 14‐deoxy‐20‐OHE, could be detected within 5 hr of the intake of supplement. With the intake of both the supplements (low and high dose), the peak level of parent 20‐OHE could be detected within 4–5 hr, and that of metabolite 14‐deoxy‐20‐OHE within 20–24 hr of intake. Both parent and metabolite could be detected for up to 20–24 and 70–72 hr of its intake. (Figure 10a,b). The estimated peak concentration of 20 OHE was 542 ng/ml after administration of Desire X and 1296 ng/ml after administration of turkesterone.

The detection window of the metabolite 14‐deoxy‐20‐OHE was longer than the 20‐OHE, which is contrary to the findings of Parr et al,^11^ wherein with the dose of 51.5 mg of 20‐OHE the detection window of metabolite 14‐deoxy‐20‐OHE was shorter than the parent compound. Recent study from Ambrosio et al.^17^on the detection and quantitation of ecdysterone in human serum shows much lower limit of detection (LOD) and limit of quantitation (LOQ) of the analytical method facilitating detection of ecdysterone post administration in the range of 0.18 to 8.2 ng/ml.

### 3.3 Prevalence study findings

**FIGURE 11:**
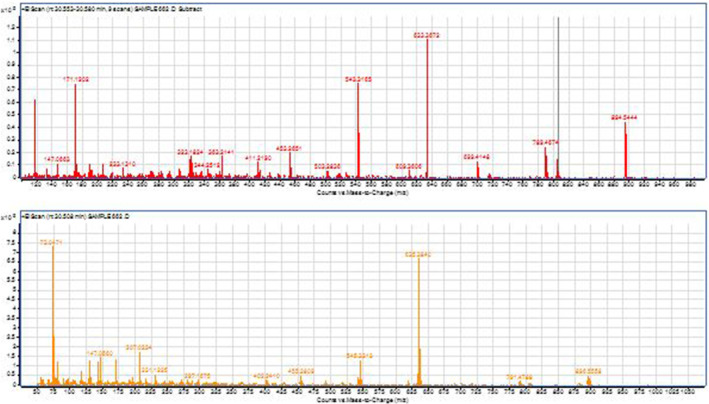
Full scan mass spectrum of 20‐OHE (upper) and 14‐deoxy‐20‐OHE (lower) by GCMSQTOF of the confirmed sample from prevalence study [Colour figure can be viewed at wileyonlinelibrary.com]

The distribution of one thousand samples is given in Figure 2. The sample preparation for the prevalence study (*n* = 1000) samples was performed according to the conventional screening procedure in antidoping laboratory. The monitoring transitions are summarized in Table 2. Nine suspicious samples were found to be showing either the parent compound or its metabolite 14‐deoxy‐20‐OHE or both of them. The confirmation procedure on GC QTOF instrument in full scan mode wherein the comparison of full scan spectrum and retention times of suspicious samples with reference standard or positive excretion study urine was performed. The four confirmed samples showed the presence of specific ions with m/z (894, 633, 543, 453, 131, and 171) for the parent compound at 30.5 min and for its metabolite 14‐deoxy‐20OHE, m/z (896, 635, 545, 171) at 30.4 min based on the ion ratios percentage, RT and mass accuracy. Minimum of three ions are used for the purpose of confirmation for both 20‐OHE and its metabolite which are presented in Tables 3 and 4 which are the theoretical values. The Δm in Tables 3 and 4 represents the mass difference between the same experimental mass for the sample and the positive control. The Figure 11 shows full scan spectrum of both parent and metabolite.

**TABLE 3:** Ion percentages, retention time, and mass accuracy calculations for the confirmation procedure for 20‐OHE performed by GCMSQTOF

Ions	Percentage %		RT (min)		Δ m	Mass accuracy (ppm)	
	**PC(+)**	**Sample**	**PC(+)**	**Sample**		**PC(+)**	**Sample**
633.3738	100	100	30.55	30.54	0.0007	5.2	4.1
894.5353	36.7	38.7			0.0006	10.1	10
543.3146	68.6	68.4			0.0004	4.23	3.5

**TABLE 4:** Ion percentage, retention time, and mass accuracy calculations for the confirmation procedure for 14‐deoxy‐20‐OHE performed by GCMSQTOF

Ions	Percentage %		RT (min)		Δ m	Mass accuracy (ppm)	
	**PC(+)**	**Sample**	**PC(+)**	**Sample**		**PC(+)**	**Sample**
635.3798	100	100	30.48	30.47	0.0006	6.6	5.82
896.5404	4.5	7.5			0.0005	5.46	6.13
545.3297	19.3	19.7			0.0000	2	2

The four confirmed cases consisted of three males(M) and one female(F), from different sports eg, aquatics (*n* = 2, 1 M/1 F), athletics (*n* = 1, M), and bodybuilding (*n* = 1, M).

## 4 CONCLUSION

This paper describes the identification of the 20‐OHE and its metabolite 14‐deoxy −20‐OHE in SCID mice urine after the administration of 20‐OHE for three consecutive days. The 20‐OHE and its metabolite, 14‐deoxy‐20‐OHE, were detectable within 24 hr. The easy accessibility to supplement via the internet allowed the purchase of 16 different supplements and quantitate 20‐OHE in five of them. Two supplements were chosen for the excretion studies (Desire X and Turkesterone), due to the presence of different concentrations of 20‐OHE., One, Desire X, unlikely to produce anabolic effects, at 0.0088 mg of 20‐OHE per capsule, while, Turkesterone, at 2.3 mg of 20‐OHE per capsule, is likely to induce anabolic effects. The urine samples were collected for up to 6 days, the parent compound and its metabolite 14‐deoxy‐20‐OHE could be detected within 2‐5 hr post administration. However, the 14‐deoxy‐ecdysterone was detectable up to 96 hr and the parent compound for up to 48 hr for the higher dose of 20‐OHE (Turkesterone, 2.3 mg of 20‐OHE/capsule). The lower dose of ecdysterone (0.0088 mg/capsule) could be detected for up to 70 hr for the metabolite 14‐deoxy‐20‐OHE and parent drug for up to 36 hr. One thousand samples from male and female athletes from different sport disciplines were analyzed for the prevalence of use for 20‐OHE, in which 4 were confirmed on further analysis. While the use seems relatively low compared to other anabolic steroids this may be explained by the current analysis of 20‐OHE only. Further studies are currently underway with other ecdysteroids, such as turkesterone, ponasterone, ajugasterone and muristerone, which are reported to be more anabolic, to determine their metabolites, window of detection, and, investigate specific uses as sport performance enhancers.
